# Forest structure, diversity, and primary production in relation to disturbance severity

**DOI:** 10.1002/ece3.6209

**Published:** 2020-04-12

**Authors:** Lisa T. Haber, Robert T. Fahey, Shea B. Wales, Nicolás Correa Pascuas, William S. Currie, Brady S. Hardiman, Christopher M. Gough

**Affiliations:** ^1^ Department of Biology Virginia Commonwealth University Richmond VA USA; ^2^ Department of Natural Resources and the Environment & Center for Environmental Sciences and Engineering University of Connecticut Storrs CT USA; ^3^ Department of Biology University of Puerto Rico at Rio Piedras San Juan PR USA; ^4^ School for Environment and Sustainability University of Michigan Ann Arbor MI USA; ^5^ Department of Forestry and Natural Resources Purdue University West Lafayette IN USA

**Keywords:** biodiversity, complexity, disturbance severity, function, primary production, structure

## Abstract

1. Differential disturbance severity effects on forest vegetation structure, species diversity, and net primary production (NPP) have been long theorized and observed. Here, we examined these factors concurrently to explore the potential for a mechanistic pathway linking disturbance severity, changes in light environment, leaf functional response, and wood NPP in a temperate hardwood forest.
2. Using a suite of measurements spanning an experimental gradient of tree mortality, we evaluated the direction and magnitude of change in vegetation structural and diversity indexes in relation to wood NPP. Informed by prior observations, we hypothesized that forest structural and species diversity changes and wood NPP would exhibit either a linear, unimodal, or threshold response in relation to disturbance severity. We expected increasing disturbance severity would progressively shift subcanopy light availability and leaf traits, thereby coupling structural and species diversity changes with primary production.
3. Linear or unimodal changes in three of four vegetation structural indexes were observed across the gradient in disturbance severity. However, disturbance‐related changes in vegetation structure were not consistently correlated with shifts in light environment, leaf traits, and wood NPP. Species diversity indexes did not change in response to rising disturbance severity.
4. We conclude that, in our study system, the sensitivity of wood NPP to rising disturbance severity is generally tied to changing vegetation structure but not species diversity. Changes in vegetation structure are inconsistently coupled with light environment and leaf traits, resulting in mixed support for our hypothesized cascade linking disturbance severity to wood NPP.

## Graphical abstract

We examined multiple metrics of vegetation structural and biological diversity concurrently to explore the potential for a mechanistic pathway linking disturbance severity, changes in light environment, leaf functional response, and wood NPP in a temperate hardwood forest. Significant linear or unimodal changes in three of four vegetation structural indexes were observed across the gradient in disturbance severity, although disturbance‐related changes in vegetation structure were not consistently correlated with shifts in light environment, leaf traits, and wood NPP. We conclude that, in our study system, the sensitivity of wood NPP to rising disturbance severity is generally tied to changing vegetation structure but not species diversity.

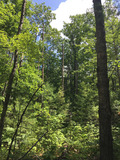

## 1 INTRODUCTION

**FIGURE 1:**
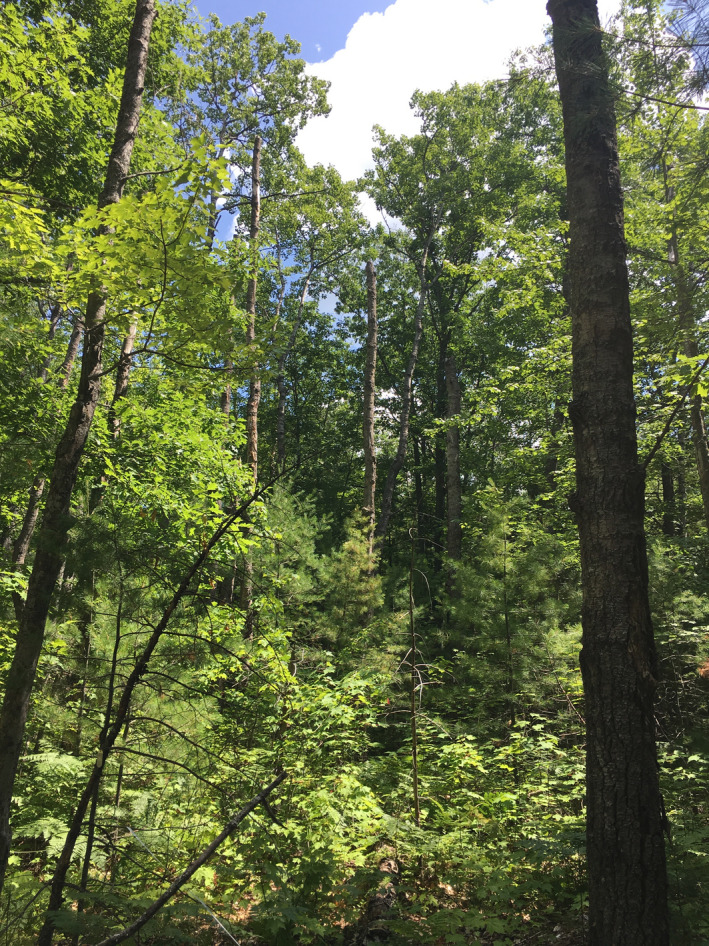
Our study system is a northern temperate mixed hardwood forest where experimental disturbance has altered vegetation structure, including the creation of deep canopy gaps

Disturbances modify forest structure (Figure 1) and, in doing so, may alter core ecosystem functions, including net primary production (NPP). Effects of disturbance severity on indexes describing forest tree species diversity and vegetation structure and, separately, on NPP have been long theorized and observed (Clements, 1916; Pickett & White, 1985). However, these effects have rarely been examined together despite evidence of forest structure–function coupling in a number of ecological contexts (Scheuermann, Nave, Fahey, Nadelhoffer, & Gough, 2018; Silva Pedro, Rammer, & Seidl, 2017). Joint investigation of forest structure–function relationships is timely as the range of disturbance severities present on temperate forest landscapes expands and, consequently, broadly reshapes plant species diversity and vegetation structure (Seidl et al., 2017; Turner, 2010) and NPP (Stuart‐Haëntjens, Curtis, Fahey, Vogel, & Gough, 2015), sometimes in surprising ways (Curtis & Gough, 2018). This widespread broadening of disturbance severity is caused by a recent proliferation of low to medium severity disturbances—those that result in partial rather than complete tree mortality—from insect pests, pathogens, and extreme weather, which in many temperate regions are outpacing increases in severe stand‐replacing disturbances (Cohen et al., 2016). Concurrent observations of changes in tree species diversity, vegetation structure, and NPP across a range of disturbance severities thus provide an opportunity to examine an understudied structure–function linkage (Fahey et al., 2016; Hardiman, Bohrer, Gough, Vogel, & Curtis, 2011).

Though rooted in different theoretical foundations, separate studies of tree species diversity, vegetation structure, and NPP suggest a similar array of responses to variable disturbance severities, implying that structural and functional changes across disturbance continua may be linked. Unimodal (Connell, 1978), linear (Hicke et al., 2012), and threshold (Tilman et al., 2001) trends in species diversity, vegetation structure, and NPP have been observed across disturbance severity gradients. For species diversity, inconsistent patterns across disturbance severity and frequency gradients have garnered considerable attention and are a source of ongoing debate (c.f. Fox, 2013; Huston, 2014), though very high levels of disturbance tend to consistently drive down diversity (Alroy, 2017; Bendix, Wiley, & Commons, 2017). Vegetation structure indexes summarizing tree distribution and dimensional heterogeneity exhibit similarly variable patterns of change across disturbance severity gradients (Hardiman et al., 2013; Sagara et al., 2018). The range of NPP responses to disturbance severity is less studied (Curtis & Gough, 2018), but observations of aquatic and terrestrial ecosystems together with model predictions suggest unimodal, linear, and threshold responses may occur in nature (Amiro et al., 2010; Stuart‐Haëntjens et al., 2015), pointing to the possibility of parallel changes in forest structure and production after disturbance.

**FIGURE 2:**
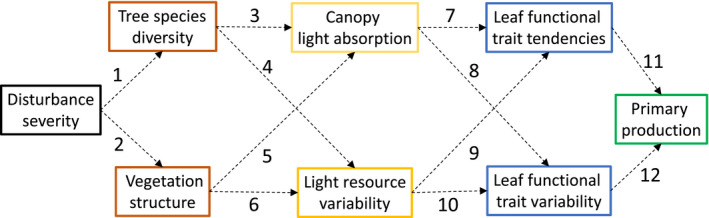
Hypothesized sequence of mechanistic linkages coupling disturbance severity and net primary production. Boxes illustrate ecological variables, while arrows represent relationships between variables. Citations indicate example prior studies where these bivariate relationships have been supported: (1) Connell, 1978; (2) Sousa, 1984; (3) Sercu et al., 2017; (4) Canham, Finzi, Pacala, & Burbank, 1994; (5) Ishii & Asano, 2010; (6) Parker & Brown, 2000; (7) Ellsworth & Reich, 1993; (8) Niinemets, 2010; (9) Rozendaal, Hurtado, & Poorter, 2006; (10) Santiago & Wright, 2007; (11) Chapin, 2003; and (12) Nicotra et al., 2010

Synchronous and mechanistically coupled changes in species diversity, vegetation structure and NPP across disturbance severity gradients could arise through a cascade of interrelated disturbance‐driven shifts in forest structure, resource availability and distribution, and leaf functional traits (Figure 2). In this framework, disturbance reshapes species diversity and vegetation structure, features closely linked with growth‐limiting resource availability and variability within canopies (Halpern & Spies, 1995). The collective production of maturing forests approaching middle stages of succession may be especially poised to benefit from moderate levels of disturbance that reallocate resources from senescent, short‐lived species to longer‐lived successors with limited resources (Odum, 1969). Resource (e.g., light) quantity and variability within canopies, both of which may increase when moderate severity disturbance augments species diversity and vegetation structural heterogeneity (Ishii & Asano, 2010; Sercu et al., 2017), ostensibly drive corresponding changes in leaf physiological or other functional traits that may in turn affect ecosystem‐scale carbon fixation (Fotis & Curtis, 2017). Though linkages between any two contiguous segments of this cascade—for example, resource environment and plant physiological response—are established, the presence of a continuous chain of cause and effect that links disturbance severity with ecosystem structure and NPP has not been investigated.

Here, we examined whether wood NPP of forest plots spanning an experimental disturbance severity gradient responded to changes in tree species diversity and vegetation structure over a subsequent 9‐year period. We also evaluated whether forest structure–function coupling in this context occurred through intermediary shifts in patterns of light availability and variability and concurrent changes in leaf physiological and morphological properties. Prior work at our site, the University of Michigan Biological Station (UMBS), focused separately on vegetation structure or production change following disturbance (Gough et al., 2013; Sagara et al., 2018). In the present study, we asked the following: (Q1) “How did species diversity and vegetation structure change along a continuum of disturbance severity?”; (Q2) “were disturbance‐driven changes in species diversity and vegetation structure correlated with availability and variability of understory light, with implications for leaf physiological and morphological traits?”; and (Q3) “were changes in species diversity and vegetation structure related to wood NPP, and was this relationship modulated by concomitant shifts in light environment and leaf traits?”. Drawing from plant physiological, community, and ecosystem ecology principles, our goal is to advance understanding of forest structure–function relationships across disturbance severity continua.

## 2 MATERIALS AND METHODS

### 2.1 Site and experiment description

This study was part of the Forest Accelerated Succession ExperimenT (FASET) at the University of Michigan Biological Station (UMBS) in northern Lower Michigan (45°35.5′N, 84°43′W). FASET was initiated in 2008 to examine biogeochemical, including carbon (C) cycling, processes following disturbance caused by age‐related senescence of aspen and birch (Nave et al., 2011). The treatment involved stem girdling all aspen (*Populus grandidentata* Michx*.* and *tremuloides* Michx.*)* and birch (*Betula papyrifera* Marshall*)* trees within a 39‐hectare area, accelerating the transition from early to middle succession in advance of that which is occurring region‐wide (Wolter & White, 2002). Prior to the experimental disturbance in 2008, the canopy was dominated by early successional aspen and birch, which broadly colonized the upper Great Lakes region following widespread clear‐cut harvesting and fire at the turn of the 20th century (Gough, Vogel, Harrold, George, & Curtis, 2007)*.* Since our experimental stem girdling, northern red oak (*Quercus rubra* L.) and red maple (*Acer rubrum* L.) have gained canopy dominance, with eastern white pine (*Pinus strobus* L.), American beech (*Fagus grandifolia* Ehrh.), sugar maple (*Acer saccharum* Marshall), striped maple (*Acer pensylvanicum* L.), and subcanopy shrub species in the genus *Amelanchier* (serviceberry) making up the remainder of abundant woody species (Fahey et al., 2016).

Our data collection centered on twice stem mapped 20 m × 20 m plots within a contiguous hectare of the FASET manipulation that spanned a disturbance severity gradient, expressed as the plot‐level percentage of tree basal area killed by girdling, from 37% to 86% (Figure S1, Table S1). The fraction of basal area lost within a plot was identical to the fraction of basal area comprised of aspen and birch, which varied across plots due to small‐scale, and putatively random, variation in these species' abundances. Prior to the experiment in 2007 and then again after manipulation‐induced tree mortality in 2015 or 2016, the spatial location of each tree stem was mapped via laser rangefinder (TruPulse 360R laser rangefinder, Laser Technology Inc.), and the diameter at breast height (DBH) and species identification were recorded for 1,589 woody stems with a DBH ≥ 1 cm in 15 of 25 plots. These plots were selected based on their high predisturbance aspen and birch abundance, thereby extending the continuum of disturbance severity beyond 60% tree mortality, the disturbance threshold at our site beyond which production steeply declined (Stuart‐Haëntjens et al., 2015). Stem map data were used to derive a suite of plot tree species diversity and structural measures as well as the annual change in live wood biomass (NPP) over the intervening 8‐ or 9‐year period. Additionally, we paired stem map data with light and leaf trait information, the methods of which are detailed below.

### 2.2 Species diversity and vegetation structure indices

From stem maps, we computed plot‐scale tree species diversity and vegetation structure metrics before (2007) and after (2015 or 2016) disturbance across the continuum of severity (Table 1). Our analysis incorporated several related but distinct species diversity and vegetation structure variables with demonstrated sensitivity to disturbance and impacts on productivity (Bourdier et al., 2016; Dănescu, Albrecht, & Bauhus, 2016). We categorized measures describing physical attributes—irrespective of tree species identity—as “vegetation structure,” in contrast to those which quantified species diversity in either a spatially explicit or agnostic way.

**TABLE 1:** Vegetation structural and species diversity metrics computed in this study

Index	Computation	Explanation of variables
Shannon's diversity index, *H* (Shannon & Weaver, 1949)	$H=-\sum_{i=1}^{S}p_{i}\ln\left(p_{i}\right)$	S: total number of species in the community $p_{i}:$ proportion of S made up of the i th species
Species mingling index, *M* (Pommerening, Gonçalves, & Rodríguez‐Soalleiro, 2011)	$M=\sum_{k=1}^{n}\frac{k}{n}m_{k}=\frac{1}{N}\sum_{i=1}^{N}M_{i}$	n: number of nearest neighboring trees analyzed per individual (4) k: number of nearest neighbors that are conspecific trees $m_{k}:$ number of trees having each possible value of the ratio $\frac{k}{n}$ N: total number of trees
Gini coefficient, *G* (Bourdier et al., 2016)	$\text{Gini}=2\frac{\sum_{i=1}^{n}ig_{i}}{\mathrm{nG}}-\frac{n+1}{n}$	$g_{i}:$ DBH of tree i G: sum of all tree diameters n: total number of trees
Coefficient of variation, *CV*	$\text{CV}=\frac{\sigma }{\mu }\times 100\%$	σ: standard deviation μ: sample mean
Diameter differentiation index, *T_d_* (Pommerening, 2002)	$T_{\mathrm{ij}}=1-\frac{\min\left({\text{DBH}}_{i},{\text{DBH}}_{j}\right)}{\max\left({\text{DBH}}_{i},{\text{DBH}}_{j}\right)}$; $T_{i}\in \left[0,1\right]$	$T_{\mathrm{ij}}:$ diameter differentiation for the i th reference tree and its nearest neighbor j (*j* = 1, 2, or 3)
Clark and Evans aggregation index, *R* (Clark & Evans, 1954)	$R=\frac{{\bar{r}}_{\text{obs}}}{E\left(r\right)}$, where $E\left(r\right)=\frac{1}{2\times \sqrt{\frac{N}{A}}}$; $R\in \left[0,2.1419\right]$	${\bar{r}}_{\text{obs}}$: mean observed distance from trees to their nearest neighbors $E\left(r\right):$ mean nearest neighbor distance in a Poisson forest with N total trees and area of A

Note

Either the original citation of the metric or a representative publication describing its use in an ecological context is provided, excluding the coefficient of variation.

For vegetation structure, we derived two spatial and two nonspatial metrics (Pommerening, 2002; Szmyt, 2014; Table 1). Because spatial arrangement of stems, irrespective of tree species, may have implications for productivity (Pacala & Deutschman, 1995; Williams, Paquette, Cavender‐Bares, Messier, & Reich, 2017), we included two metrics that contain tree location information in addition to two that capture size difference. The spatially agnostic measures were as follows: the coefficient of variation of stem diameter (CV DBH), a relative measure of variability in stem sizes within plots; and the Gini coefficient of DBH (G), a dimensionless measure of stem size inequality. The spatially explicit structural metrics were as follows: the diameter differentiation index (*T_d_*), a nearest neighbor metric expressing the average stem size difference between neighboring trees; and the aggregation index of Clark and Evans (*R*), with *R* = 1 indicating a completely random distribution of stems (a Poisson process), *R* > 1 a tendency toward regular spacing among stems, and *R* < 1 a clustered spatial pattern in stem locations. To account for edge effects between adjacent plots that might influence clumping patterns of stems, the Donnelly correction (Donnelly, 1978) was applied to the Clark and Evans index computation. Two tree species diversity measures were derived: the nonspatial Shannon species diversity index (H) and a nearest neighbor spatial metric, the species mingling index (M). Only stems that were alive in 2007 and/or at the time of remeasurement (in 2015 or 2016) were included in the derivation of these indexes, all of which were computed using R statistical software (R Core Team, 2017).

### 2.3 Aboveground wood net primary production

We derived 8‐ or 9‐year aboveground wood NPP across the disturbance severity gradient from total plot live wood mass increment between 2007 and 2015 or 2016. We first estimated plot wood mass before and after disturbance for all live stems with DBH ≥ 1 cm using region‐specific allometric equations relating DBH to wood mass (Perala & Alban, 1994) and then divided this total increment by the number of intervening years to obtain an annual production value. To compare relative responses to disturbance of plot wood NPP, irrespective of initial production, we report the difference in individual plot wood NPP from the 15‐plot mean wood NPP. This difference is expressed as plot fraction of departure from mean wood NPP (hereafter, NPP_dep_) and was calculated by dividing the wood NPP of each plot by mean wood NPP, quantity minus one.

### 2.4 Leaf area index

We assessed peak leaf area index (LAI) in 2016 through optical imaging of the canopy. Hemispherical skyward‐facing images at plot centers were taken at 1 m above the forest floor under diffuse light using a leveled camera with a 180° fisheye lens. Images were registered using ImageJ (version 1.51; Schneider, Rasband, & Eliceiri, 2012), and estimates of LAI were derived using Gap Light Analyzer (Version 2.0; Frazer, Canham, & Lertzman, 1999) software with MINIMUM thresholding applied, as this algorithm is suitable for canopies with gaps (Inoue, Yamamoto, & Mizoue, 2011).

### 2.5 The fraction of photosynthetically active radiation absorbed by canopies

To evaluate whether changes in species diversity and vegetation structure corresponded with canopy light interception, we quantified the fraction of photosynthetically active radiation (fPAR) absorbed by the canopies of each plot at peak LAI in 2016. We used an AccuPAR LP‐80 ceptometer (Decagon Devices Inc) to measure ground‐level PAR along a 2 m × 2 m gridded 400‐m^2^ area within each plot for a total of 100 measurements. Concurrent above‐canopy PAR measurements were obtained from an Apogee SQ‐110 quantum sensor (Apogee Instruments Inc.) positioned on a nearby (< 200 m) meteorological tower, and ground and above‐canopy PAR matched to the closest (≤ 5 min) datum for the derivation of fPAR. Measurements were attempted under clear sky conditions between the hours of 11:30 a.m. and 4:00 p.m. from mid‐July to early August 2016, but intermittent cloud cover in 9 plots forced the omission of 1% – 32% of total PAR measurements in these plots.

### 2.6 Leaf physiology and morphology

We examined the means and variability of leaf physiological and morphological characteristics across the disturbance severity continuum during peak leaf out (mid‐July to mid‐August) in 2016. To capture representative variation along vertical and horizontal canopy axes, we established 1‐m^2^ quadrats at 0, 2, 4, and 6 m from the center of our plots along the four cardinal axes. Two leaves, irrespective of species, that came closest to intercepting the vertical axis at the center of the quadrat at 1 m and 3 m canopy height were selected for measurements. When the leaves of woody species were absent from a quadrat, bracken fern *(Pteridium aquilinum* L.), the most prominent subcanopy herbaceous species, was sampled if present. Though up to 26 leaves per plot could be sampled using our protocol, actual sample size varied from a minimum of 6 to a maximum of 20 leaves because of vegetation gaps.

For each sampled leaf, we measured photosynthetic capacity of light‐saturated leaves (*A*_sat_) and apparent quantum yield using a LI‐6400XT Portable Photosynthesis System (LI‐COR Incorporated, Lincoln, Nebraska, USA). *A*_sat_ was the stable maximum rate at which light‐saturated (2,000 µmol photons m^−2^ s^−1^) leaves assimilated carbon dioxide (µmol CO_2_ m^−2^ s^‐1^). Apparent quantum yield of photosynthesis (*q*) values was obtained for 1‐m leaves through light response curve model fitting using R code for nonlinear least squares regression of a nonrectangular hyperbola (Heberling & Fridley, 2013). Our models used 10 measurements for fitting curves and derived *q* with the following parameters: incoming PAR, photosynthetic rate (*A*_net_), maximum photosynthetic rate (*A*_max_), daytime dark respiration rate (*A*_net_ at PAR = 0), and a dimensionless curve convexity parameter. Although modeled light response curve fits were attempted for all 97 leaves collected at 1 m height, 18 of the leaves' models failed to converge on a closed solution; thus, we present *q* results derived from 79 statistically significant (*α* = 0.05) curves.

We characterized leaf morphology as leaf mass per area (LMA), a commonly measured leaf trait useful in distinguishing shade‐ from sun‐adapted leaves, and one which is sensitive to disturbance‐driven changes in subcanopy light regime (Poorter, Niinemets, Poorter, Wright, & Villar, 2009). Individual leaf area was determined using a LI‐3100C Area Meter (LI‐COR Incorporated). Pine needles and deciduous broadleaf specimens were each included in analysis and scanned at the appropriate resolution (0.1 mm^2^ and 1 mm^2^, respectively). Leaves were subsequently dried at 60°C for 48 hr and then weighed to calculate leaf mass per area.

### 2.7 Statistical analysis

To evaluate whether disturbance severity shifts species diversity and vegetation structure, and consequently initiates a cascade of changes in fPAR, leaf physiology and morphology, and ultimately wood NPP, we conducted a series of regression analyses based on a priori expectations of the cause‐and‐effect order and shape of these relationships. For all response variables, we evaluated three disturbance severity–response relationships grounded in prior published observations: a linear null model (Hicke et al., 2012); a unimodal quadratic relationship adhering to the intermediate disturbance hypothesis (Connell, 1978); and, a threshold model reflecting an abrupt nonlinear transition or break point (Stuart‐Haëntjens et al., 2015). All three model fits were attempted, and the model that had significance at *α* = 0.10 with the lowest Akaike information criterion corrected for small sample size (AICc) value was chosen. To enable comparison across models and to guard against inflation of explanatory power, we report the adjusted *r* ^2^ value for each selected model. A synthesis of disturbance–structure–function relationships was conducted to determine whether the postulated ordered cascade of effects—with disturbance shifting vegetation structure and species diversity, light capture, leaf physiology, and finally NPP—was supported statistically (Figure 2). Linear and nonlinear modeling and AICc computation were conducted using SigmaPlot 13.0 (Systat Software Inc.). In addition, we employed Levene's test for equality of variance in fPAR, LMA, *A*_sat_, and *q* values across the disturbance severity continuum using R.

## 3 RESULTS

### 3.1 Disturbance severity and aboveground wood net primary production

**FIGURE 3:**
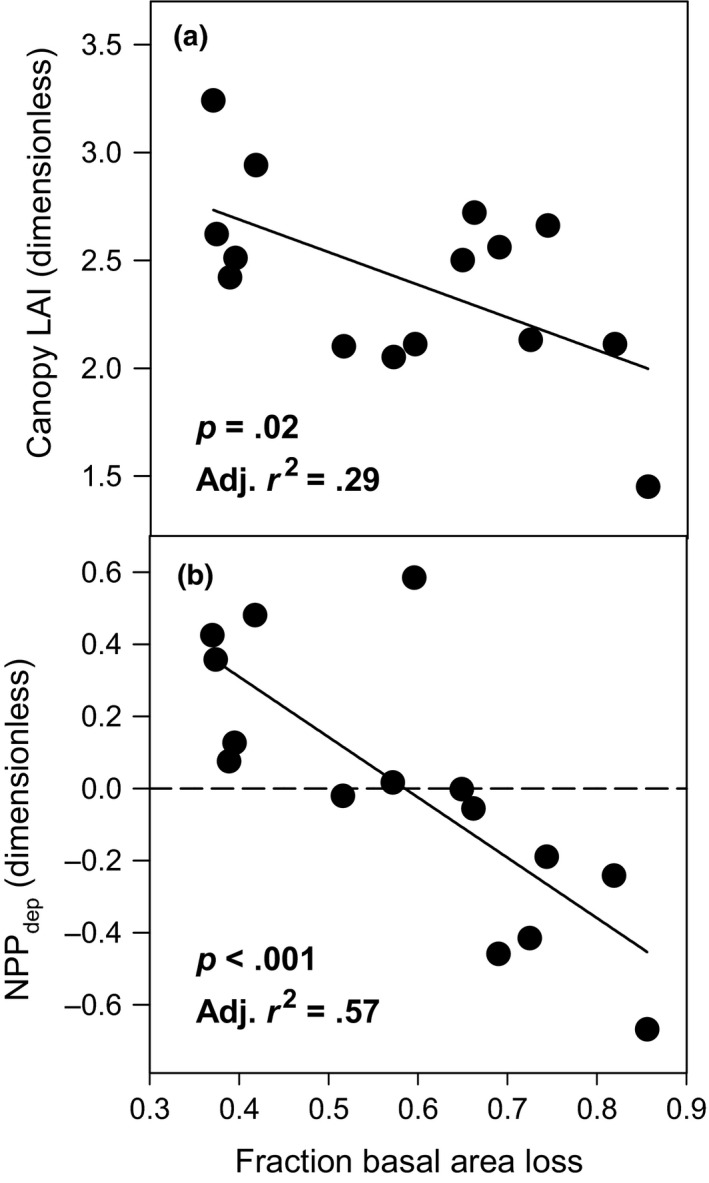
Leaf area index (a, LAI) and fraction of departure from mean wood NPP (b, NPP_dep_) in relation to disturbance severity expressed as the fraction of basal area loss

Establishing continuity across studies, our observations of declining LAI and plot‐level fraction of departure from mean wood NPP (NPP_dep_) with increasing tree basal area mortality (Figure 3a,b) were consistent with those previously observed for our site (Stuart‐Haëntjens et al., 2015), while extending the upper limit of disturbance severity by nearly 20%. Comparable to nearby plots (Stuart‐Haëntjens et al., 2015), we found NPP_dep_ declined when basal area mortality exceeded approximately 60% (Figure 3b). In contrast to the nonlinear threshold response observed by Stuart‐Haëntjens et al. (2015) at 4 or 5 years following disturbance, at 8 or 9 years postdisturbance we observed a linear decline in canopy LAI and NPP_dep_ as basal area losses (i.e., as disturbance severity) increased (*p* = .02, Adj. *r* ^2^ = .29, AICc = −24.3; *p* < .001, Adj. *r* ^2^ = .57, AICc = −37.1).

### 3.2 Study Q1: Species diversity and vegetation structure

**FIGURE 4:**
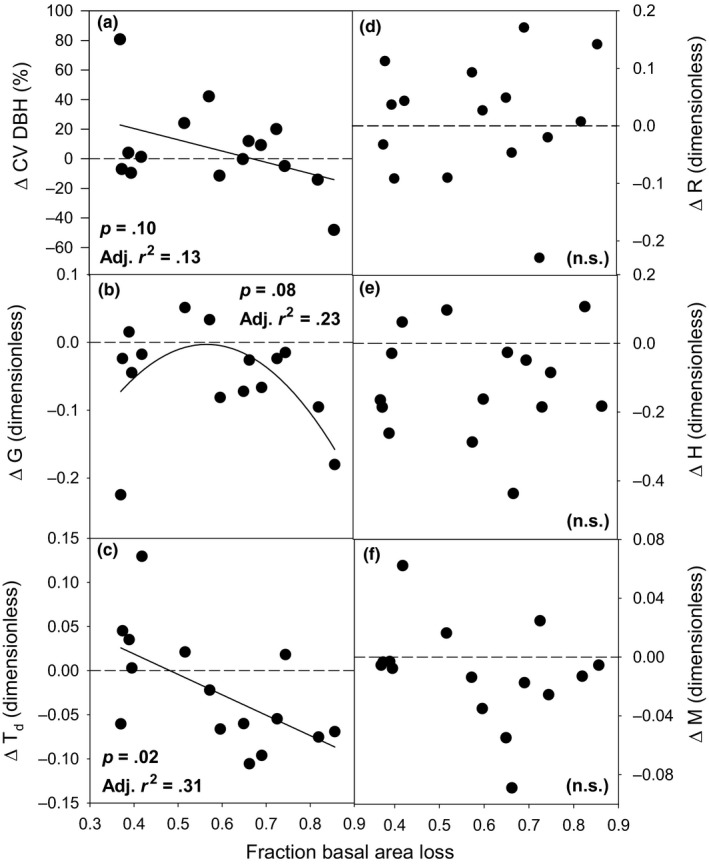
Vegetation structure and species diversity changes (Δ) across a disturbance severity continuum expressed as fraction of basal area loss. Vegetation structure indices are as follows: the coefficient of variation of DBH (a, CV DBH), the Gini coefficient of DBH (b, *G*), the diameter differentiation index (c, *T_d_*), and the Clark and Evans aggregation index (d, *R*). Diversity indices are as follows: Shannon's diversity index (e, *H*) and the species mingling index (f, *M*)

We observed significant but variable changes in most vegetation structural but not species diversity measures across the disturbance severity continuum. Three of four vegetation structure metrics, none of which correlated with aspen and birch basal area across plots before the experiment, exhibited significant changes (Δ) with rising disturbance severity (Figure 4a–d). The coefficient of variation in DBH declined weakly and linearly with rising disturbance severity (*p* = .10, Adj. *r* ^2^ = .13; AICc = 104.8), while spatially explicit Δ*T_d_* showed a stronger linear decline (*p* = .02, Adj. *r* ^2^ = .31; AICc = −81.8). ΔG followed a weakly unimodal trend across the disturbance severity gradient, with lower DBH inequalities (i.e., greater homogeneity) occurring at low and high disturbance severities and peak values between 50% and 60% basal area senesced (*p* = .08, Adj. *r* ^2^ = .23; AICc = −73.4), corresponding with the disturbance level at which NPP_dep_ began to decline. The shift in Clark and Evans aggregation index (Δ*R*) did not exhibit a significant relationship with disturbance severity (linear model: *p* = .84, Adj. *r* ^2^ = −.07, AICc = −61.2). Neither species diversity measure, Shannon's diversity, or the spatially explicit species mingling index (Figure 4e,f) changed across the disturbance severity gradient (linear model results: *p* = .91, *p* = .26, respectively).

### 3.3 Study Q2: Canopy light interception, leaf morphology, and physiology

**FIGURE 5:**
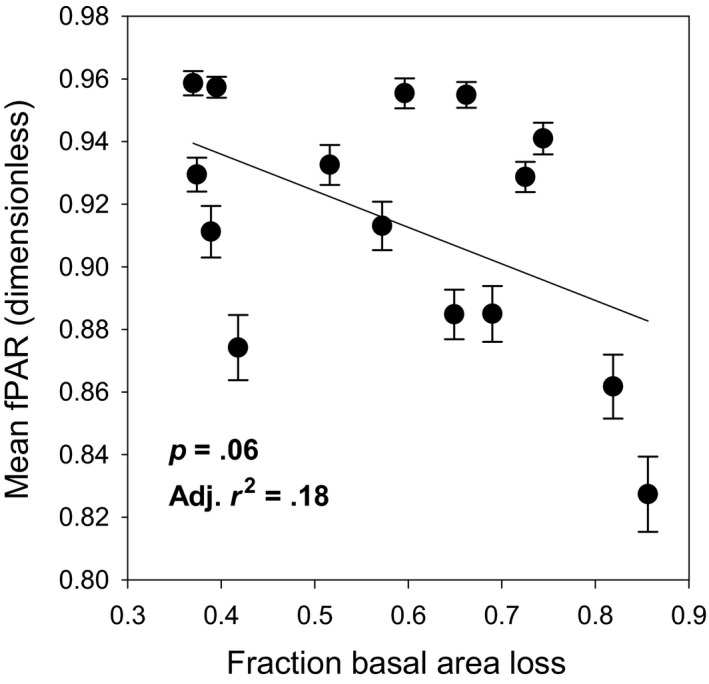
The fraction of photosynthetically active radiation (fPAR) absorbed by the canopy in relation to disturbance severity expressed as the fraction basal area loss. Means ± 1 *SE*

We observed a significant negative linear relationship between canopy light interception and disturbance severity (*p* = .06, Adj. *r* ^2^ = .18, AICc = −93.6; Figure 5), mirroring the trend in declining NPP_dep_ (Figure 3b) with rising disturbance. Variance in fPAR (expressed as CV fPAR) exhibited no significant relationship with disturbance severity when tested for linear, unimodal, or threshold model fits (*p* = .14, *p* = .13, and *p* = .31, respectively), although evidence of nonconstant variance was found in fPAR measurements across the disturbance severity continuum via Levene's test (*p* < .001; Table 2).

**TABLE 2:** Statistical test results for means and equality of variance (Levene's test) for canopy light interception (fPAR) and leaf physiological (maximum rate of light‐saturated photosynthesis, *A*_sat_; apparent quantum yield of photosynthesis, *q*) and morphological (LMA) parameters

	Light	Physiology and morphology		
	fPAR	LMA	*A*_sat_	*q*
Mean versus disturbance severity	***p* = .04** (unimodal)	*p* = .69	*p* = .29	*p* = .29
Levene's test	***p* < .001**	*p* = .28	***p* < .001**	*p* = .11

Note

Significance was determined at *α* = 0.10 for all tests, and significant *p*‐values are shown in bold. Where no significant fit was found among candidate linear, unimodal, and threshold relationships, *p*‐values for linear models are reported.

Rising disturbance severity reduced the spatial variability of some physiological but not morphological leaf traits and had no effect on mean values at the plot scale. Mean plot LMA (including deciduous broadleaf and evergreen needleleaf species) did not exhibit a significant pattern of change across the disturbance severity continuum, nor did either leaf physiological parameter examined (*A*_sat_ and *q*, data not shown). However, coefficients of variation (CV) for *A*_sat_, *q*, and LMA declined at high disturbance severities, with maximal values occurring below 50% basal area loss. Levene's test for equality of variances provided strong evidence for heteroscedasticity in *A*_sat_ values across plots (*p* < .001; Table 2), though not for the apparent quantum yield of photosynthesis (*q*; *p* = .11) or for LMA (*p* = .28).

### 3.4 Study Q3: Cascading disturbance–structure–production interactions

**FIGURE 6:**
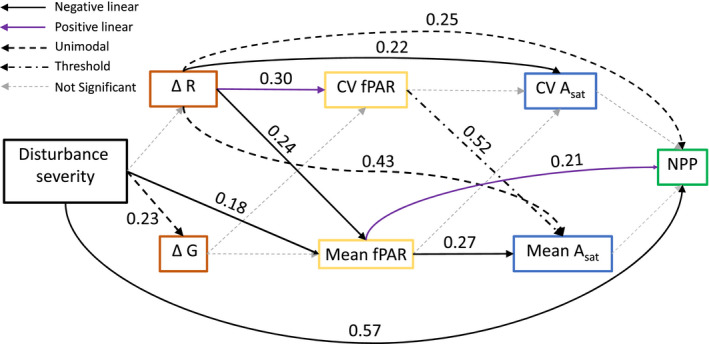
Illustration of an interconnected disturbance–structure–function cascade for two vegetation structural metrics: the aggregation index of Clark and Evans (Δ*R*) and the Gini index of DBH (Δ*G*). Δ*R*, though not correlated with disturbance severity, exhibited either unimodal or linear relationships with all five downstream variables tested. In contrast, Δ*G*, though related unimodally to disturbance severity, did not relate to canopy light capture (fPAR), leaf‐level physiology (*A*_sat_), or fraction of departure from mean wood net primary production (NPP). To enable direct comparison of different models across this proposed mechanistic pathway, adjusted *r* ^2^ values are provided for each variable linkage, and models were selected based on the lowest Akaike information criterion correction for small sample size (AICc) scores. *p*‐values and AICc scores are provided in Table S2. Only one threshold relationship was retained across variables tested

We examined whether changes over time in forest structure—shaped by or independent of disturbance—are linked to wood NPP through an interrelated cascade of relationships coupling structure with fPAR, leaf physiology, and NPP_dep_. We focus on two vegetation structure metrics: the Gini index of DBH (ΔG), exhibiting a significant change with rising disturbance severity (*p* = .08, Adj. *r* ^2^ = .23, AICc = −73.4), and the Clark and Evans aggregation index (ΔR), which did not change with disturbance but was significantly unimodally related to NPP_dep_ (*p* = .07, Adj. *r* ^2^ = .25, AICc = −26.1; Figure 6). Even though Δ*G* followed a unimodal distribution across the disturbance continuum, this measure of vegetation structure exhibited no relationship with fPAR, CV *A*_sat_, or wood NPP_dep_, resulting in a broken chain between disturbance‐driven changes in stem diameter distribution and downstream mechanisms hypothesized to affect NPP. In contrast, ΔR was unrelated to disturbance severity, but its change over the 8‐ to 9‐year study period was correlated via negative linear, unimodal, and positive linear models with all four intermediary variables (mean and CV fPAR, mean and CV *A*_sat_) in our hypothetical mechanistic pathway (Figures 2 and 6) and directly with NPP_dep_.

## 4 DISCUSSION

We found forest structural indexes describing vegetation structure but not species diversity changed across a disturbance severity gradient spanning 37%–86% tree basal area loss, but that wood NPP was not related to forest structural shifts mediated by disturbance. Most of the vegetation structural indexes that we examined declined at high disturbance severities and, contrary to some prior results (Buckling, Kassen, Bell, & Rainey, 2000; Budke, Jarenkow, & de Oliveira‐Filho, 2010), species diversity indexes exhibited no change with increasing disturbance severity. Disturbance‐driven changes in vegetation structure, though prevalent, were not linked with wood NPP. Instead, we found changes over time in the Clark and Evans aggregation index—the only vegetation structural index unrelated to disturbance severity—correlated with wood NPP, indicating forest plots trending toward structural uniformity, independent of disturbance, had lower production.

Similar to the mixed relationships broadly reported in the literature (Hughes, Byrnes, Kimbro, & Stachowicz, 2007; Mackey & Currie, 2001), we found the effect of disturbance severity on forest structure measures was mixed, with unimodal or linear responses in three of four vegetation structure measures but no pattern of effects on tree species diversity. The decline of three different vegetation structural measures at high levels of disturbance severity points to a multifaceted increase in vegetation structural uniformity at high levels of disturbance. Consistent with our findings, moderate disturbance severity may increase vegetation structural heterogeneity (Seidl, Rammer, & Spies, 2014), but such responses are variable (Biswas & Mallik, 2010) possibly because of differences in predisturbance material legacies, community composition, successional stage, and vegetation distribution (Dietze & Matthes, 2014). Though we observed no relationship between species diversity indexes and disturbance severity, our null findings are aligned with recent observations (Hughes et al., 2007; Mackey & Currie, 2001). Ecological theory—including the intermediate disturbance hypothesis (IDH)—posits peak species diversity in the moderate or intermediate range of disturbance frequency and intensity (Connell, 1978; Huston, 2014), but, as with vegetation structure, empirical support for a universal unimodal relationship is mixed (Mackey & Currie, 2001). In our forest ecosystem, vegetation structure may show greater sensitivity to changing disturbance severity because tree species diversity was already low prior to disturbance (mean tree species richness = 7.4). Additionally, at our study site within FASET, the loss of a single plant functional type (fast‐growing, short‐lived early successional trees)—simulating successional change region‐wide—was consistent across the entire manipulation. Taken together, the mixed significance and shape of forest structure–disturbance severity relationships that we observed reinforce the importance of applying system‐dependent context to the interpretation of ecological theory (Huston, 2014).

Disturbance severity modified the subcanopy light environment and exerted mixed effects on leaf traits. Deeper canopy light penetration is widely observed at higher disturbance severities (Fauset et al., 2017; Turton & Siegenthaler, 2004) as is increased light spatial homogeneity as canopies become more uniformly open (Chazdon & Fetcher, 1984). We incorrectly anticipated that at high disturbance severities, a more homogenous and enriched subcanopy light environment would consistently augment leaf trait uniformity and promote sun leaf physiology and morphology. Significantly different variances in plot‐level fPAR across the disturbance continuum, despite a concomitant decline in fPAR at high disturbance severity, may explain why leaf morphology and one of two leaf physiological parameters (*q*, but not *A*_sat_) remained steady with rising disturbance severity. Underlying our expectation of covarying light environment and leaf trait properties are observations linking growth‐limiting resource availability and variability across topographic, successional, and disturbance gradients with the means and variability of plant traits (Herben, Klimešová, & Chytrý, 2018; Wilfahrt, 2018). Moreover, earlier observations from our experimental site showed significant changes in subcanopy leaf trait profiles 4 years after disturbance (Stuart‐Haëntjens et al., 2015). Our results instead show that changes in subcanopy light environment and leaf traits 9 years after disturbance were not proportional to tree mortality, suggesting that leaf physiological and morphological traits may have returned to their predisturbance means, indicative of functional resilience (Hillebrand et al., 2017).

Despite strong pair‐wise relationships between many variables, we did not observe a continuous mechanistic cascade coupling disturbance severity with NPP. Though an interconnected cause‐and‐effect chain was not detected, bivariate relationships along our proposed cascade are supported by prior observations linking: disturbance severity and forest structure (Sousa, 1984); vegetation structure and light environment (Ishii & Asano, 2010); light environment and leaf physiology and morphology (Niinemets, 2010); and leaf physiology and morphology and primary production (Chapin, 2003). We may not have observed a mechanistic linkage joining vegetation structure and production because relationships within every segment of the hypothesized cascade (e.g., disturbance severity versus vegetation structure; Figure 2) were often not significant or only weakly significant and, accordingly, unlikely to carry forward to NPP (Figure 6). Both our small sample size of 15 plots and the inherent variability within our study ecosystem yielded uncertainty in our analysis, and we caution that strong evidence for or against relationships among variables was not consistently detected. We also may not have captured the most functionally important mediating processes—in our case, light environment and leaf physiological and morphological traits—connecting vegetation structure and wood NPP. However, prior work from our site (Stuart‐Haëntjens et al., 2015) and other forests (Atkins, Fahey, Hardiman, & Gough, 2018) demonstrates that light is a growth‐limiting resource tied to species diversity and vegetation structure and, separately, that leaf photosynthetic traits can be predictors of ecosystem‐level production (Wang et al., 2015). Our inconclusive findings bolster recent pleas for manipulative experiments aimed at identifying the mechanisms linking ecosystem structure and function following disturbance (Hillebrand et al., 2017; Hooper et al., 2005).

Though disturbance‐shaped vegetation structure was not coupled with wood NPP, changes in stem arrangement over time—independent of disturbance—had effects on primary production. Wood NPP was greatest when the Clark and Evans stem aggregation index was stable over time (Δ*R* = 0), with plots trending toward a more clumped stem arrangement (Δ*R* < 0) or a more ordered pattern (Δ*R* > 0) exhibiting the lowest relative NPP_dep_. A tendency toward more ordered or uniform stem arrangements corresponded with less canopy light absorbed and reduced variability in *A*_sat_. Vegetation structure, even when not altered by recent disturbance, can exert a strong influence over production (Hardiman et al., 2011). However, numerous unresolved questions center on understanding which vegetation structural features are most closely tied to production and whether they change over time and across ecosystems. The principal vegetation structural determinant of forest production may change as ecosystem development unfolds over decades to centuries (Silva Pedro et al., 2017) and may differ among plant functional types (Scheuermann et al., 2018). Additionally, the successional context of ecosystems—in our study case, a forest in transition from early to middle stages—differentially influences the production response to disturbance. Our findings would likely not have been the same in a fast‐growing, early successional forest, nor in an old‐growth forest with fewer subcanopy trees poised to benefit from disturbance‐mediated release. Advancing understanding of structure–function relationships will require nuanced consideration of the way vegetation structural and species diversity attributes are shaped and, in turn, shape production, with attention to multiple scales of organization and time required to understand the breadth of patterns found in nature.

## 5 CONCLUSIONS

Determining how and why disturbance—which is increasing in frequency and extent globally—will modify forest ecosystem structure and functioning remains a grand challenge because of the complexity, variability, and dynamic nature of these core ecosystem properties. Our results, though mixed, suggest that several interrelated linkages exist in our study ecosystem between disturbance severity, vegetation structure (but not species diversity), subcanopy light environment and leaf traits, and NPP. However, our findings also reveal a lack of complete continuity linking disturbance to structure and functional change through the mediating effects of changing resource environment and leaf physiology and morphology. We conclude that additional investigation of multiple ecosystems at various stages of development is needed to identify which structural changes owing to disturbance affect core ecosystem functions such as primary production.

## CONFLICT OF INTEREST

None declared.

## AUTHOR CONTRIBUTIONS

**Lisa Haber**: Conceptualization (equal); data curation (lead); formal analysis (lead); investigation (equal); methodology (equal); visualization (lead); writing – original draft (lead); writing – review and editing (equal). **Robert T. Fahey**: Conceptualization (equal); investigation (equal); methodology (equal); writing – review and editing (supporting). **Shea B. Wales**: Data curation (supporting); investigation (supporting); writing – review and editing (supporting). **Nicolas Correa Pascuas**: Data curation (supporting); investigation (supporting). **Willliam Currie:** Conceptualization (supporting); data curation (equal); funding acquisition (supporting); investigation (supporting); methodology (supporting); writing – review and editing (equal). **Brady S. Hardiman**: Formal analysis (supporting); validation (equal); writing – review and editing (equal). **Christopher Gough**: Conceptualization (lead); formal analysis (supporting); funding acquisition (lead); investigation (equal); methodology (equal); project administration (lead); resources (lead); supervision (lead); visualization (supporting); writing – original draft (supporting); writing – review and editing (equal).

## Supporting information

Fig S1

Table S1‐S2

## Data Availability

Raw data for all analyses from 2007 and 2015–2016, as well as code used to run structural analyses, are available through the Figshare digital repository: 10.6084/m9.figshare.11935197.
