# Supplementary figures and images for: Forest structure, diversity, and primary production in relation to disturbance severity
Source: Ecol Evol. 2020 Apr 12;10(10):4419–30. doi: 10.1002/ece3.6209 (PMC7246213; doi:10.1002/ece3.6209)

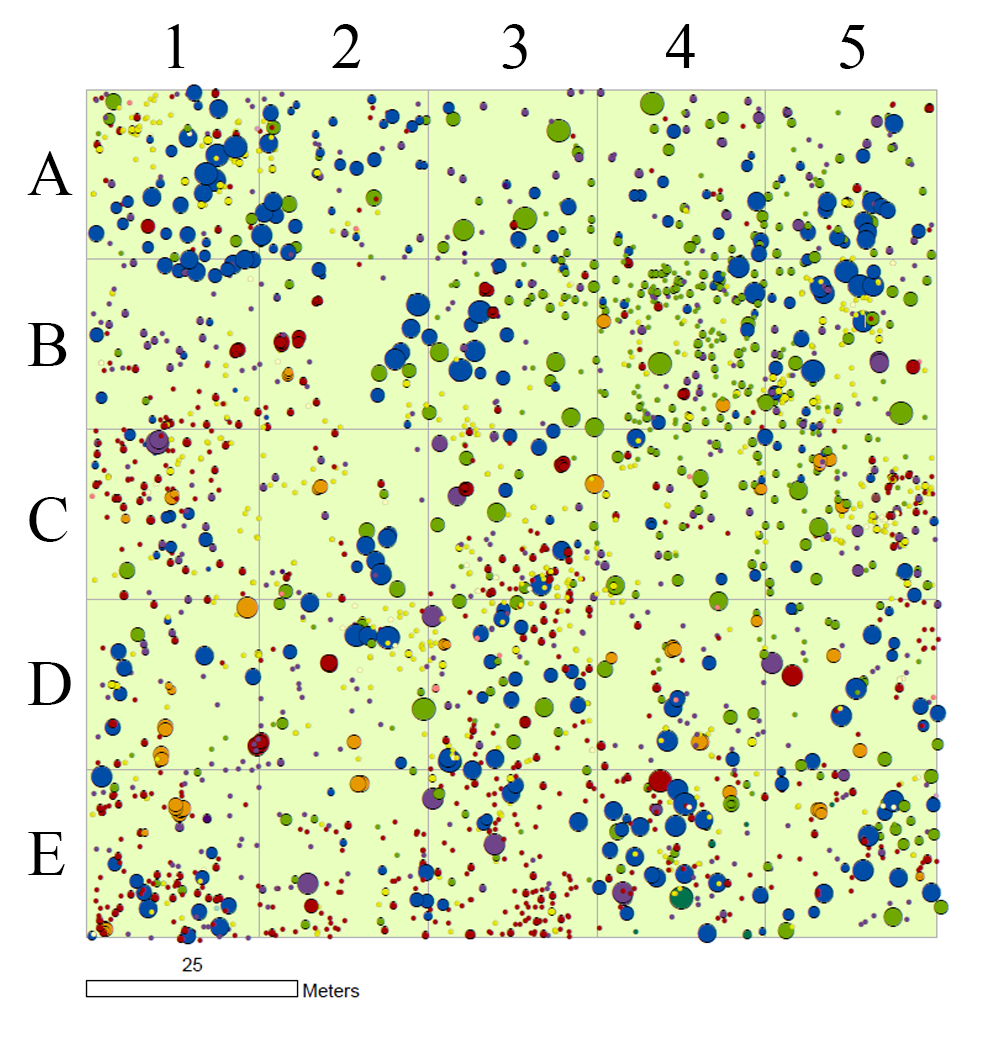

Supplement: Supplementary file 1 — Fig S1 [file ECE3-10-4419-s001.png]
