# Supplementary material for: Forest structure, diversity, and primary production in relation to disturbance severity
Source: Ecol Evol. 2020 Apr 12;10(10):4419–30. doi: 10.1002/ece3.6209 (PMC7246213; doi:10.1002/ece3.6209)
Supplement: Supplementary file 2 — Table S1‐S2 [file ECE3-10-4419-s002.docx]

**Supplemental Table 1.** Wood net primary production (NPP) and plot-level fraction of difference from mean NPP (NPP_dep_) estimates for the interval from summer 2007 (pre-disturbance) to summer 2015 or 2016 (post-disturbance). Biomass (kg C) increment was derived from stem growth measurements and site- and species-specific allometries; these values have been scaled up to the hectare from the 20 m x 20 m plot measurement. NPP_dep_ was obtained by normalizing each plot’s biomass increment to the mean increment across all 15 plots, in order to allow for meaningful comparison across plots with heterogeneous pre-disturbance biomass.

| **Plot ID** | **Fraction of Basal Area Loss (dimensionless)** | **Wood NPP**  **(metric tonnes C ha^-1^)** | **NPP_dep_ (dimensionless)** |
| --- | --- | --- | --- |
| A3 | 0.37 | 4.08 | 0.42 |
| C3 | 0.37 | 3.88 | 0.36 |
| C5 | 0.39 | 3.08 | 0.07 |
| B4 | 0.40 | 3.22 | 0.13 |
| D2 | 0.42 | 4.24 | 0.48 |
| B3 | 0.52 | 3.15 | -0.02 |
| D5 | 0.57 | 3.27 | 0.02 |
| B5 | 0.60 | 5.10 | 0.58 |
| D3 | 0.65 | 3.21 | 0.00 |
| E5 | 0.66 | 3.04 | -0.06 |
| D4 | 0.69 | 1.74 | -0.46 |
| C2 | 0.73 | 1.88 | -0.42 |
| A5 | 0.74 | 2.61 | -0.19 |
| A1 | 0.82 | 2.44 | -0.24 |
| D1 | 0.86 | 1.06 | -0.67 |

**Supplemental Table 2.** Statistical test results for significant relationships identified among variables of interest in Figure 6: the aggregation index of Clark and Evans (ΔR), mean plot-level canopy light interception (fPAR), the Gini index of DBH (ΔG), fraction of departure from mean net primary production (NPP_dep_), and maximum rate of light-saturated photosynthesis (A_sat_). Final models were selected from candidates (linear, unimodal, and threshold) below the significance level α = 0.10 based on lowest AICc score.

| **Variable 1** | **Variable 2** | **Model** | ***p*** | **Adjusted r^2^** | **AICc** |
| --- | --- | --- | --- | --- | --- |
| Disturbance Severity | ΔG | unimodal | 0.08 | 0.23 | -73.4 |
|  | Mean fPAR | linear | 0.06 | 0.18 | -93.6 |
|  | NPP_dep_ | linear | < 0.001 | 0.57 | -37.1 |
| ΔR | CV fPAR | linear | 0.02 | 0.30 | 60.1 |
|  | Mean fPAR | linear | 0.04 | 0.24 | -94.7 |
|  | CV A_sat_ | linear | 0.04 | 0.22 | 116.8 |
|  | Mean A_sat_ | unimodal | 0.01 | 0.43 | 26.7 |
|  | NPP_dep_ | unimodal | 0.07 | 0.25 | -26.1 |
| CV fPAR | Mean A_sat_ | threshold | 0.01 | 0.52 | 27.6 |
| Mean fPAR | Mean A_sat_ | linear | 0.03 | 0.27 | 28.0 |
|  | NPP_dep_ | linear | 0.05 | 0.21 | -28.1 |
